# Endotoxin removal therapy with Polymyxin B immobilized fiber column as a COVID-19-bedside strategy protocol for endotoxic shock

**DOI:** 10.3389/fneph.2022.847305

**Published:** 2022-08-03

**Authors:** Silvia De Rosa, Monica Zanella, Sara Samoni, Claudio Ronco

**Affiliations:** ^1^ Department of Anesthesiology and Intensive Care, San Bortolo Hospital, Vicenza, Italy; ^2^ International Renal Research Institute of Vicenza, Vicenza, Italy; ^3^ Department of Nephrology, Dialysis and Transplantation, San Bortolo Hospital, Vicenza, Italy; ^4^ Department of Nephrology and Dialysis, ASST Lariana, S. Anna Hospital, Como, Italy; ^5^ Department of Medicine, University of Padova, Padova, Italy

**Keywords:** coronavirus disease 2019, endotoxic shock, hemoperfusion, renal replacement therapy group, sequential extracorporeal therapy

## Abstract

Endotoxin -induced sepsis is a leading cause of ICU mortality. From 1994 to the present, PMX-HP has been available as an adjuvant therapy for endotoxin removal and immunomodulation. The efficacy and usefulness of this therapy have been demonstrated for more than a quarter of a century and are partially supported by clinical studies. However, it appears that selected subgroups of patients with endotoxic shock and with appropriate timing could benefit. Endotoxemia may be involved in the pathophysiology of COVID-19, based on enterocyte dysfunction and malabsorptive syndrome. Due to the characteristics of the microbiota, Gram-negative bacteria or their fragments (i.e., endotoxin) may translocate into the systemic circulation leading to inflammatory activation, immune dysfunction, and sepsis. In addition, patients with severe forms of COVID-19 are at risk of superimposed infections. Endotoxemia can arise due to the translocation of Gram-negative bacteria or their fragments from the gut barrier. According to the most updated evidence available from large randomized trials, septic shock patients with MODS > 9 and EA levels ranging from 0.6 to 0.9 are those who may benefit the most from PMX‐HP treatment in terms of improvement of survival. As shown in a previous publication, we believe that similarly to the source control, microbiological cultures, and antibiotics administration, EA evaluation at regular intervals, and the targeted use of PMX‐HP could be lifesaving and adequate within the golden hour for the diagnosis and treatment of endotoxic shock. In our center, we applied a diagnostic-clinical flowchart also for endotoxic shock related to COVID-19.

## Introduction

The World Health Organization (WHO) named the virus 2019 novel coronavirus (2019-nCoV) on January 12, 2020, and declared a global public health emergency on January 30, 2020 (1). The rate of serious illness of severe acute respiratory syndrome coronavirus 2 (SARS-CoV-2) infection, revealed by epidemiological data, is equal to 25%, and although the lungs are the main organs affected, the kidney is also one of the main organs affected in the seriously ill (2). In more severe infections, the percentage of patients requiring renal replacement therapy (RRT) increased to 5.2-25% (3, 4).

Although continuous renal replacement therapy (CRRT) is the most widely used blood purification modality in clinical practice, severe coronavirus disease 19 (COVID-19) patients with sepsis and acute respiratory distress syndrome (ARDS) can be treated with plasma hemoperfusion/adsorption to eliminate inflammatory mediators (5). The emergence of COVID-19 cases limited hospital resources, including the selection of RRT modality or hemoperfusion, consumable supplies, personnel, and the emergence of unexpected complications such as a hypercoagulable state leading to frequent clotting and wastage of RRT cartridges, and made it necessary to carefully assess available resources on a daily basis. COVID-19-associated acute kidney injury (AKI): consensus report of the 25^th^ Acute Disease Quality Initiative (ADQI) Workgroup suggested considering adjustments to RRT modality, indications, anticoagulation, and dose as part of a local response to an imbalance in supply and/or demand to conserve scarce resources and deliver effective therapy to the greatest number of patients (not graded) (6). In addition, the ADQI consensus group suggested projecting a workforce planning and response to a surge in COVID-19 cases for trained RRT nursing support, ensuring the availability of relevant human resources (6, 7). Several promising drugs are being tested in clinical trials for COVID‐19, but so far there is no established therapy. In our hospital, we formed an RRT Group to ensure optimal care for patients requiring blood purification therapies. Particularly, COVID-19-infected patients with confirmed endotoxic shock were treated with Polymyxin B-immobilized hemoperfusion (PMX-HP) based on our institutional diagnostic-clinical flowchart (8). In this article, we report our experience with the formation and organization of the RRT group and our diagnostic-clinical flowchart applied for endotoxic shock related to COVID-19.

## Endotoxemia in COVID-19 and polymyxin B hemoperfusion

The SARS-CoV-2 virus enters target cells *via* the angiotensin-converting enzyme-2 (ACE-2) receptor, which is expressed in enterocytes in the ileum and colon (9, 10), potentially leading to endotoxin translocation from the intestinal tract which might exacerbate the severity of COVID-19. The breakdown in the intestinal barrier might lead to endotoxin translocation independently of Gram-negative bacteremia (11, 12), suggesting that a “leaky” or dysfunctional barrier might contribute to endotoxin translocation. Additionally, an adverse ventilation strategy (13) can cause systemic translocation of endotoxins. This translocation might contribute to sepsis-associated AKI due to systemic hypotension, direct renal vasoconstriction, activation of vasoactive hormones, and activation of inflammatory pathways (14). Endotoxin activity is a valuable biomarker of disease severity. Although in critically ill patients, there is a tight correlation between endotoxin levels and severity of the septic shock, organ dysfunction, and the risk of death (15), it is not well investigated in COVID-19 patients. Recent reports suggested a high incidence of secondary bacterial infection in severe COVID-19 patients, varying from 58 to 80.3% (16–18). A COVID-19 study found that 6% of patients experienced septic shock during hospitalization (19). In addition, COVID-19 patients with septic shock complications seem to have a much higher death rate than those without septic shock (20).

Endotoxin removal during antibiotic therapy is critical to prevent inflammation, tissue death, and endotoxic shock. Endotoxic shock is *an emergency condition* where appropriate and prompt administration of antimicrobial therapy and proper source control may improve the patients’ chance of survival. Endotoxin removal can be performed by using PMX-HP which has been safely used for the treatment of septic shock since 1994. The immobilized polymyxin B molecules bind the lipid A portion of the endotoxin *via* ionic and hydrophobic interactions. PMX-HP allows not only endotoxin removal but is also an immunomodulatory device. The immunostimulatory effects and the cellular elements inducing immunomodulatory and anti-apoptotic effects with PMX-HP have been demonstrated (21). Although there is some evidence to support the efficacy of PMX-HP treatment in selected populations with endotoxic shock, the use of PMX-HP is questioned by a few studies showing no benefit (22) to support the routine use of PMX-HP for the treatment of patients with endotoxic shock. In a cohort of severe critically ill patients admitted to the intensive care unit (ICU) with SARS-CoV-2 infection, who developed septic shock during the ICU stay and recorded in the EUPHAS2 registry, PMX-HP was associated with organ function recovery over 5 days afterward, mainly due to respiratory and hemodynamic improvement (23). In addition to general agreement on personalized indications for this adjunct therapy, timing problems persist for its initiation. Recent guidelines for the surviving sepsis campaign, albeit a weak recommendation, have taken a stance against this therapy. However, the mechanism of action of PMX-HP is not fully understood (24).

## Appropriate management of extracorporeal blood purification therapies across the septic shock pathway

**Figure 1:**
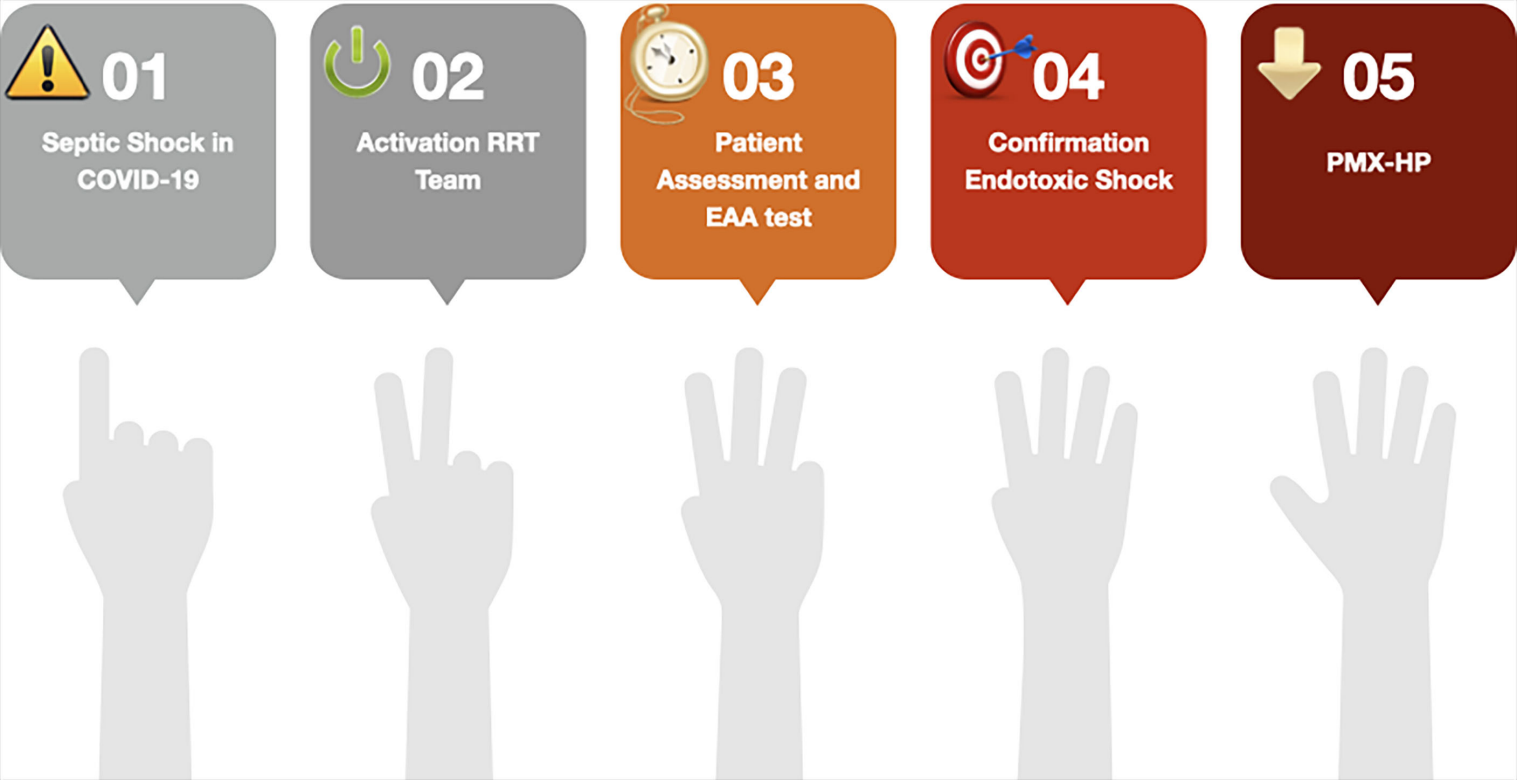
The five steps time-dependent approach for endotoxic shock in coronavirus disease 19 (COVID-19). Acronym: EAA, Endotoxin Activity Assay; RRT, Renal Replacement Therapy; PMX-HP, Polymyxin B hemoperfusion.

PMX-HP can be life-saving when treating bacterial infections but is often used inappropriately. Although most clinicians are aware of the existence of a golden hour for septic shock, most underestimate this problem in their hospitals. Clinicians should always optimize antimicrobial management and source control to maximize the clinical outcome of the patients and apply appropriately this bridge therapy. The necessity of formalized systematic approaches to the optimization of extracorporeal blood purification therapies (EBPT) in the setting of septic shock has become increasingly urgent. We proposed in **Figure 1** the time-dependent five steps approach for the identification of endotoxic shock supported by an efficient and well-trained multidisciplinary team.

In our ICU, we have a strategy just applied before the COVID-19 pandemic, for the correct use of EBPT and based on followed strategies:

1. Communication and education;
2. Updating local epidemiological data stratifying them for specific settings;
3. Start and choice of treatment using a severity-driven approach;
4. Drafting of local algorithms/flowchart;
5. Avoid redundant prescriptions;
6. Not being impulsive in starting EBPT;
7. Strict collaboration with nephrology laboratory in daily practice;
8. Being aware of pharmacokinetics/pharmacodynamics (PK/PD) issues for antibiotics during EBPT;
9. Creating a multidisciplinary team (RRT Group).

## Renal replacement therapy team

With the development of EBPT in the ICU setting and the subsequent establishment of Critical Care Nephrology as a new medical specialty, nephrologists and intensivists have started to collaborate in the management of critically ill patients (25). This is particularly true in our hospital where the “Vicenza model” has been born, thus being the multidisciplinary approach to critically ill patients in the standard of practice.

Before the COVID-19 pandemic, our diagnostic-clinical flowchart for clinically diagnosed septic patients gathered different specialists to the patient’s bedside to timely and appropriately diagnose and manage sepsis and its complications. This approach has been maintained also in COVID-19 patients. In life-threatening conditions, such as sepsis and COVID-19, the systematization of interventions through a diagnostic-clinical flowchart is crucial to standardize the clinical practice according to the updated evidence and promptly intervene, avoiding time loss.

For this purpose, we created an RRT team to provide EBPT support in COVID-19 patients 24/24 hours 7/7 days. It is composed of nephrologists and intensivists with expertise in EBPT in critically ill patients, trained nurses, and biologists of the Team Lab. The RRT team is activated when there is a clinical diagnosis of septic shock in the emergency department or a medical/surgical department or the ICU. The intensivist and the nephrologist expert in EBPT, based on the patient’s clinical signs and their biological markers, can prescribe PMX-HP treatment and, if specific indications are present, they can also decide to start CRRT after PMX-HP [KDIGO]. The RRT team assesses the patient over time to promptly and continuously adjust the EBPT, based on the patient’s actual needs. A dynamic prescription is particularly required in septic and/or COVID-19 patients in which the clinical picture can rapidly vary and a sequential extracorporeal therapy in sepsis (SETS) could be advocated.

## The diagnostic-clinical flowchart

In our center, critically ill patients with a clinical diagnosis of sepsis and septic shock follow a clinical diagnostic and therapeutic pathway that provides for early diagnosis and early identification of a subgroup of patients with endotoxic shock who benefit, after the first-hour bundle, of PMX-HP. The same logic has been applied to the patient population with COVID-19-related pneumonia, which manifests as bacterial co-infection, as reported in the literature (26, 27).

Beyond the pathophysiological rationale for the use of PMX‐HP for endotoxin removal, the precise clinical indication for its initiation is widely debated in the literature.

In our ICU, in line with current guidelines on sepsis management (28), the early identification (ideally within one hour of recognition) and the appropriate management (i.e. antimicrobials administration, adequate fluid resuscitation, and application of vasopressor therapies) in the initial hours after the development of sepsis is routinely performed (28), also in COVID-19 patients. Blood endotoxin activity (EA) is measured by the Endotoxin Activity Assay (EAA™) (Spectral Medical Inc, Toronto, ON, Canada), which is a rapid (30 minutes) *in vitro* test that assesses neutrophil reaction to endotoxin *via* a chemiluminescent reaction, performed by our Team Lab.

According to recent literature, EA ≥ 0.4 is associated with a high risk of sepsis and worse clinical outcome both at ICU and hospital discharge (15).

In our center, EA is routinely used to identify a specific patient population that may benefit from the PMX-HP application. Precisely, EA is performed when there is a clinical diagnosis of septic shock in the emergency department or a medical/surgical department after running the first-hour bundle. In addition, we perform EA also to monitor clinical conditions from baseline until 120 hours from the start of treatment. Although Endotoxin may have a role in the pathophysiology of COVID-19-associated infectious complications, its removal should be further investigated in large studies (23). According to the most up-to-date evidence available from large randomized trials, patients with septic shock with a MODS > 9 and an EA between 0.6 and 0.9 are the most likely to benefit from PMX-HP treatment in terms of survival improvement. The treatment protocol used 2 sessions of 2 h PMX-HP for 2 consecutive days. The vascular access that we currently use is a double-lumen catheter inserted through the central vein by Seldinger’s method. The blood flow rate is set at 120–150 mL/min. Heparin infusion is the standard anticoagulation, and in the case of heparin contraindication, a non-anticoagulation strategy is applied. However, circuit problems (29) related to thrombotic complications and coagulation disorder have been encountered and highlighted in patients with COVID-19 (30, 31).

In our center, MODS and Sequential Organ Failure Assessment (SOFA) scores are used to assess organ dysfunction in critically ill patients, including those with septic and endotoxic shock.

**Figure 2:**
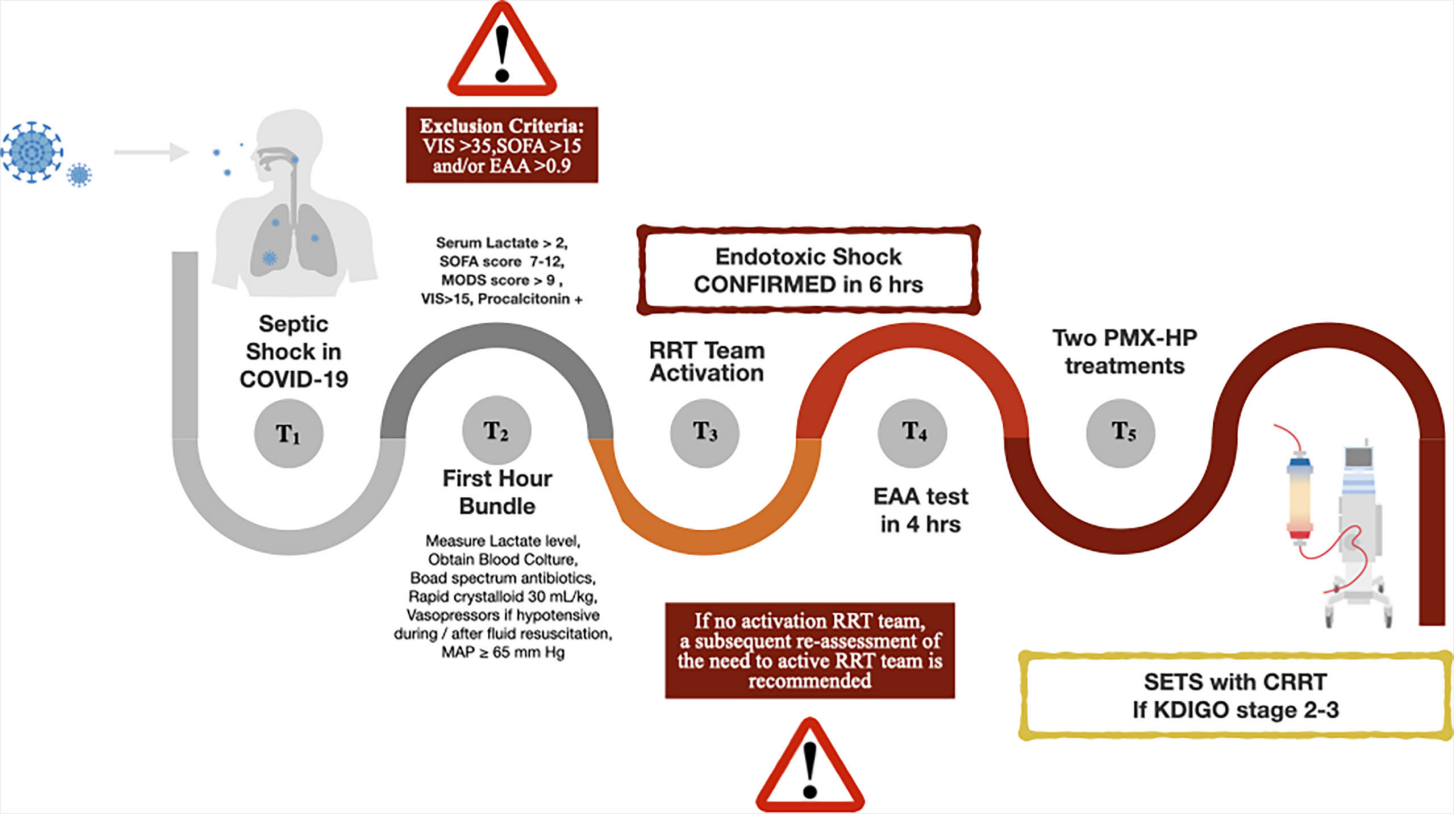
Diagnostic-clinical flowchart. Acronym: COVID-19, the coronavirus disease 2019; SOFA, Sequential Organ Failure Assessment score; MODS, Multiple Organ Dysfunction Syndrome; VIS, Vasoactive Inotropic Score; MAP, Mean Arterial Pressure; RRT, Renal Replacement Therapy; EAA, Endotoxin Activity Assay; PMX-HP, Polymyxin B hemoperfusion; SETS, Sequential Extracorporeal Therapy in Sepsis; CRRT, Continuous Renal Replacement Therapy; KDIGO, Kidney Disease: Improving Global Outcomes.

Our diagnostic-clinical flowchart is shown in **Figure 2**. As we have shown previously, based on our experience, we strongly suggest starting extracorporeal endotoxin removal within 4 hours after source control and starting antibiotic therapy (8). Based on accurate monitoring and biological markers, adjust prescription and appropriate delivery of what we defined as SETS is guaranteed (8). When organ failure develops, extracorporeal therapies may support replacing or supporting the function of several organs such as the heart, kidney, liver, and lungs. If AKI Kidney Disease: Improving Global Outcomes (KDIGO) stage 2-3 is present, we start RRT to support renal function after the first PMX-HP treatment. A second treatment with PMX-HP is recommended. The further and evident severe unresponsive shock (Vasoactive-Inotropic Score (VIS) >35) and SOFA >15) and/or a high level of EA (higher than 0.9) should be carefully evaluated and should corroborate the extracorporeal endotoxin removal initiation.

## Conclusion

In conclusion, in our center, we performed endotoxin removal therapy using PMX-HP as a COVID-19-bedside strategy protocol for endotoxic shock after a prompt and appropriate timing and selection of patients after the first-hour bundle and in case of unresponsive endotoxic shock. We found that selected patients could have some advantages from this adjunct therapy. Further studies are needed to confirm this strategy in COVID-19 patients. In addition, our next step will be to test the flowchart in future trials to place a role in endotoxin activity assessment and consequently, early, extracorporeal endotoxin removal by Polymyxin B hemoperfusion in clinical practice.

## Data availability statement

The original contributions presented in the study are included in the article/Supplementary Material. Further inquiries can be directed to the corresponding author.

## Author contributions

SD, SS and MZ have substantially contributed to the conception of the work, drafting the work or revising it critically for important intellectual content. CR has finally approved the version to be published. All authors read and approved the final manuscript.

## Conflict of interest

The authors declare that the research was conducted in the absence of any commercial or financial relationships that could be construed as a potential conflict of interest.

## Publisher’s note

All claims expressed in this article are solely those of the authors and do not necessarily represent those of their affiliated organizations, or those of the publisher, the editors and the reviewers. Any product that may be evaluated in this article, or claim that may be made by its manufacturer, is not guaranteed or endorsed by the publisher.
